# Cholera—Its Prevention and Treatment

**Published:** 1873-08-15

**Authors:** 


### MEDICAL EXAMINER.

*A Semi-Monthly Journal of Medical Sciences.*

EDITED BY

N. S. DAVIS, M. D., AND F. H. DAVIS, M. D.

Chicago, August 15th, 1873.

### EDITORIAL.

### CHOLERA—ITS PREVENTION AND TREATMENT.

We do not intend to enter upon any extended discussion of this subject at present. In the July number of the Nashville *Journal of Medicine and Surgery* the senior editor calls attention to diet as a preventative of cholera. He says, "Vegetable and fruit eaters alone die of cholera." In reference to the prevalence of cholera in Nashville in 1849, 1850, 1854, and 1866, he says, "No one who abstained from fruits, vegetables, and *animal products*, died of the disease." And again, since the recent epidemic in that city, he repeats the assertion that "Cholera would not prey upon one who had no fruits, vegetables, nor animal products in his stomach." While we were seriously casting about to see what would be left for a hungry man to live on, after excluding all "fruits, vegetables, and animal products" from his stomach, our eye caught the last paragraph of the editorial, in which our friend Bowling proposes to show that "one brave, good man, with five assistants, did drive—literally drive—cholera out of the negro villages around our city (Nashville), when it was at its worst. But they carried hope in their faces, medicine in one hand, and *chickens* in the other; but ne'er a vegetable, ne'er a time!"

To talk about preventing cholera by excluding from a man's stomach all "fruits, vegetables, and animal products," and then tell us that some good, brave man had literally driven the disease out of town by a hopeful countenance, aided by medicine in one hand and *chickens* in the other, is to mix things almost as badly as we sometimes mix ancient and modern languages in writing prescriptions.

Does not our confrere of Nashville regard chickens as animal products? And if he does, what did that brave, good man carry them in one hand for? Was it merely that they might aid the effects of the *hope* in his own face, by crowing here and there?

Seriously, however, we hope our much esteemed friend Bowling will lose no time in giving us a full account of just what diet he regards as safe for human beings to eat during the season when cholera is prevalent. We were not fortunate enough to see or notice what he published on this subject in either 1850 or 1854; and the subject is one of so much importance that we hope he will favor the profession with a more detailed statement of his recent experience, both in regard to the prevention and cure of this much dreaded disease.

We have anxiously watched our exchanges, especially those from the Mississippi and Ohio valleys, where the cholera has been prevailing during the present summer, in the hope that we might find something new and more efficient in the treatment of the disease. But, aside from the limited use of quinia, morphia, and atropia, hypodermically, we notice nothing worthy of mention that is not already familiar to our readers. We think the hypodermic injection of quinia, in moderate doses, in addition to the treatment we have heretofore recommended by the mouth and rectum, would be of much value. But the use of morphia, either alone or combined with atropia, hypodermically, should not be resorted to without great caution, especially in the stage of collapse, or its near approach.

After all that has been written on the subject, our main reliance in the treatment of cholera must be in the early adoption of such measures as are calculated to overcome the irritability and perverted action of the mucous membrane, and to destroy whatever poison or germ may be supposed to exist as the exciting cause of the disease. Anodyne and astringent injections into the rectum, after every passage; small doses of calomel and morphia, *immediately* after every act of vomiting; one-quarter of a grain of carbolic acid, in solution with a little glycerine and camphorated tincture opium, every hour at first, and later every two hours; with mustard sinapisms over the whole length of the spine and epigastrium, and entire rest, constitute the most efficient treatment for the active, violent stage of genuine cholera. The addition of hypodermic injections of quinia would probably aid the other remedies. As the system becomes drained of its fluids, towards the end of the active stage, animal broth, well salted, given alternately with strong coffee, at frequent intervals, but in table-spoonful doses, should be given for replenishment and support.

